# The First Cottage Hospital

**Published:** 1924-11

**Authors:** 


### CORRESPONDENCE.

### The First Cottage Hospital.

*To the Editor of* The Hospital and Health Review.

Sir,—I wonder if you or any of your readers could tell me the date of the foundation of the first cottage hospital and which hospital that was ? I have an impression that the first institution of the kind was opened in the middle of last century, but I am unable to find any information on the subject.

S. T.

[A cottage hospital was opened at Warwick as far back as 1823, but was that the first ?—*Editor*, The Hospital and Health Review.]

